# Comprehensive analysis of alternative splicing in *Rosa roxburghii* Tratt reveals its role in flavonoid synthesis

**DOI:** 10.3389/fpls.2025.1627126

**Published:** 2025-07-11

**Authors:** Yanlin An, Jiahua Wu, Yani Chen, Shize Li

**Affiliations:** ^1^ School of Food Engineering, Moutai Institute, Renhuai, Guizhou, China; ^2^ College of Life Sciences, Guizhou University, Guiyang, China

**Keywords:** alternative splicing, flavonoid synthesis, *Rosa roxburghii* Tratt, molecular mechanism, WGCNA

## Abstract

**Introduction:**

Alternative splicing (AS) plays an important role in the synthesis of plant metabolites. Chestnut rose is a fruit with rich metabolites and health benefits. However, the role of AS in its metabolite synthesis is rarely reported.

**Methods:**

The transcriptome data of eight tissues of *Rosa roxburghii* were analyzed by using Trimmomatic, Hisat2 and StringTie software. AStalavista tool was used to identify alternative splicing (AS) events, which were further analyzed with IGV browser and other tools. The WGCNA package in R software was applied to construct co-expression networks. AS events were validated by RT-qPCR, and the *RrActin* was used as an internal control to analyze the transcription expression pattern.

**Results:**

In this study, the AS landscape were characterized in different tissues of chestnut rose. The data showed that 8586 genes could undergo AS and a total of 49,523 AS events were generated. Among them, tissue-specific AS genes were found in leaves, flowers and fruits. The content of flavonoids in the samples was detected and WGCNA analysis was performed with the AS genes. Five key modules of AS genes related to flavonoid synthesis were identified, and *4CL*, *ANR*, *DFR*, *MYB* and other AS genes were validated by PCR and sequencing. In addition, qRT-PCR analysis revealed that the expression level of basic helix-loop-helix (*bHLH*) transcription factor AS transcript was higher than its full-length transcript, and it was highly expressed in FR1 and significantly correlated with flavonoids.

**Discussion:**

Our research identified AS events in different tissues of chestnut rose and revealed their important functions in flavonoid synthesis. This study provides the basis for the molecular mechanism of flavonoids in chestnut rose.

## 1 Introduction

Chestnut rose (*Rosa roxburghii* Tratt.), a member of the *Rosaceae* family, is a fruit native to the southwestern region of China (Huang et al., 2014). It derives its name from its golden color at maturity and its surface, which is densely covered with small fleshy thorns. It contains abundant vitamin C, superoxide dismutase (SOD), flavonoids, and other bioactive compounds known for their antioxidant, immune-enhancing, blood sugar and lipid lowering, and cardiovascular protection (Hou et al., 2019, 2021). Due to its unique health effects, it has gained popularity among consumers in China in recent years. At present, research on chestnut rose mainly focuses on its functional components, while scientific exploration of its molecular mechanisms began relatively late. For example, studies have compared the levels of catechin, myricetin, and SOD in two varieties of chestnut rose from different regions (Hou et al., 2020). UHPLC-IM-QTOF and UPLC-QQQ techniques were used to analyze free and bound phenolic compounds in the fruit (Xu et al., 2023). Recently, the genomes of both chestnut rose and its seedless variant, with sizes of 504 M and 981 M respectively, were assembled using nanopore and Hi-C sequencing technologies (Zong et al., 2024a), thereby advancing molecular research on the species. In another study, 168 transcription factors related to the vitamin C content were identified in chestnut rose, including RrHY5H and RrZIP9, which regulate gene expression (Su et al., 2024). We also identified the OVATE gene family and demonstrated its important role in plant growth and development (An et al., 2024).

Flavonoids refer to a group of compounds derived from a 2-phenylchromenone backbone, which has multiple health benefits (Dias et al., 2021; Shen et al., 2022). While the molecular mechanisms underlying flavonoid synthesis have been extensively studied in plants such as strawberry (Warner et al., 2021), Ginkgo biloba (Guo et al., 2023), and tea plant (Shi et al., 2023), similar studies on chestnut rose remain limited. In tea plants, for example, the chalcone synthase (CHS) gene has been shown to regulate flavonoid synthesis and respond to light stress (Li et al., 2024). In addition, research on peony petals revealed that chalcone synthase regulates flavonoid synthesis through ubiquitination (Gu et al., 2019). The dihydroflavanol 4-reductase (DFR) gene has also been shown to regulate the synthesis of delphinidin, a type of flavonoid, in the genus *Iris*. In terms of transcriptional regulation, the MYB–bHLH–WDR complex has been shown to regulate flavonoid synthesis. For example, *FaMYB5* and *FaMYB10* can regulate the accumulation of anthocyanins by modulating the expression of *LAR* and *ANR*, respectively (Yue et al., 2023). Similarly, a genome-wide analysis of the bHLH gene family in *Spatholobus suberectus* identified *SsbHLH112* as a regulator of flavonoid synthesis (Qin et al., 2025).

Alternative splicing (AS) is a process by which a single gene produces different mRNA isoforms through different patterns of splicing (Syed et al., 2012; Wang et al., 2015). Walter Gilbert first proposed the concept of AS in 1978, and in 1980, David Baltimore discovered the first AS gene, IgM, in mice (Cartegni et al., 2002; Ast, 2004). Since then, AS has become a widely studied topic due to its important functional roles. AS events can be classified into four main types: intron retention (IR), alternative 3’ splice sites (A3SS), alternative 5’ splice sites (A5SS), and exon skipping (ES), depending on the nature of the splicing site and exon combination (Wang et al., 2024). While ES is the most common AS type in animals, IR predominates in plants (Filichkin et al., 2010; Tao et al., 2024). In plants, AS has been shown to be involved in many life processes such as growth and development, metabolite synthesis, and stress resistance (Mi et al., 2024; Qiao et al., 2024; Tian et al., 2024; Zong et al., 2024b). For example, in poplar, *PtRD26* undergoes AS to generate an isoform, PtRD26^IR^, with intron retention, which interacts with multiple NAC transcription factors to negatively regulate leaf senescence (Wang et al., 2021). In wheat, *Pm4* produces two transcripts with distinct protein domains; both are required for resistance to powdery mildew and are localized in the endoplasmic reticulum (Sánchez-Martín et al., 2021). Zhong et al. constructed a pan -transcriptome using data from 11 rice varieties subjected to low-temperature treatment to analyze genes involved in transcriptional regulation. They found that AS plays a significant role in transcriptional regulation in response to low temperature stress (Zhong et al., 2024). The synthesis of metabolites in chestnut rose is the result of multiple gene regulation. As an important post-transcriptional regulation mechanism, the role of AS in the synthesis of chestnut rose metabolites remains largely unexplored.

This study conducted a comprehensive analysis of AS using transcriptome sequencing data from different tissues of chestnut rose. We observed a large number of AS genes in different tissues and identified a total of 49,523 AS events, with many AS genes showing tissue-specific expression. By integrating flavonoid content data with AS gene expression in a weighted gene co-expression network analysis (WGCNA), we identified numerous AS genes associated with the synthesis of naringenin, catechin, and quercitrin. The existence of these AS transcripts involved in the flavonoid synthesis pathway was also verified based on PCR and sequencing, such as *RrDFR*, *Rr4CL*, and *RrANR*. Additionally, the expression patterns of AS genes *RrbHLH* and *RrGT* across eight tissues were validated by qRT-PCR, suggesting their functions in flavonoid synthesis. These results provide new insights into the regulatory role of AS in flavonoid synthesis in *Rosa roxburghii* Tratt and also reveal the multiple functions of AS in plant molecular regulation.

## 2 Materials and methods

### 2.1 Plant materials

In total, eight samples representing different tissues and developmental stages were collected from wild *Rosa roxburghii* Tratt grown under natural conditions in Renhuai City, Guizhou Province, China (E:106.38、N:27.85). The sampled tissues were as follows: FL1 (Flower bud), FL2 (Flower), LF (Leaf), FR1 (the first sampling of the fruit at the growth stage), FR2 (the second sampling of the fruit at the growth stage), FR3 (the third sampling of the fruit at the growth stage), FR4 (the fourth sampling of the fruit at the growth stage), and FR5 (the fifth collection of fruits approaching maturity) (**Supplementary Figure S1**). Three biological replicates were collected for eight samples All samples were immediately frozen in liquid nitrogen and stored at − 80°C until further use.

### 2.2 Transcriptome data analysis

RNA-seq data were downloaded from the Genome Sequence Archive (https://ngdc.cncb.ac.cn/gsa) under accession number CRA017453. Raw sequencing reads were quality-filtered using Trimmomatic.v0.36, and the remaining paired-end reads were used for subsequent analyses (Bolger et al., 2014). The filtered reads were mapped to the reference genome using Hisat2.v2.2.1 software with default parameters (Pertea et al., 2016), generating sequence alignment map (SAM) files. These SAM files were converted to BAM format and used for transcript assembly with StringTiev.2.2.1. Gene transfer format (GTF) files from each sample were then merged using the “–merge” option in StringTie (Mi et al., 2021). The merged GTF file served as a reference for reassembling transcripts across all samples using the -e-B parameters in StringTie, thereby ensuring uniform transcript annotations and quantification of gene expression levels.

### 2.3 Identification and analysis of alternative splicing

AS events were identified using the AStalavista tool on a localized Linux platform (Foissac and Sammeth, 2007). The merged GTF file was used as input, and AS events were detected using the -t asta parameter. Four major types of AS events were extracted from the output files and counted, including intron retention (IR; AS code: 1^2-,0), exon skipping (ES, AS code: 1-2^, 0), alternative 3′ splice site (A3SS, AS code: 1-,2-), and alternative 5′ splice site (A5SS, AS code: 1^,2^). An AS event was considered valid only if its corresponding transcript had an FPKM value greater than 1 across all three biological replicates. AS events were visualized using the IGV browser (Thorvaldsdóttir et al., 2013). An Up-set Venn diagram was generated using an online tool (BioLadder, https://www.bioladder.cn/web/#/pro/index). KEGG enrichment analysis of AS genes was performed using TBtools and visualized in R using the ggplot2 package (Wickham, 2011).

Transcripts per million (TPM) values for all samples were generated using StringTie to evaluate transcript expression levels. To reduce the impact of low-abundance transcripts in downstream analyses, transcripts with TPM values <1 in any sample group were excluded. AS events were defined as present when at least two transcripts of a given gene remained after filtering. Differential expression analysis was performed using the R package DEGseq.v1.52.0 (Wang et al., 2010). Transcripts with a fold change > 2 and a *q-*value < 0.01 were considered as differentially expressed transcripts (DETs). Genes exhibiting AS and containing at least one DET were classified as differentially expressed AS genes s (DAGs).

### 2.4 Detection of flavonoid substances

A total of 50 mg of each tissue sample was accurately weighed, ground in liquid nitrogen, and mixed with 300 μL of methanol containing an internal standard. All samples were sonicated in ice water for 10 minutes and then incubated at -20°C for 1 hour. After centrifugation at 13,000 rpm for 15 minutes, the supernatants were collected for further analysis. Flavonoid profiling was conducted using liquid chromatography–mass spectrometry (LC-MS) on a Waters ACQUITY I-Class PLUS Ultra High Performance Liquid Phase Tandem Waters Xevo G2-XS QT of high-resolution Acquity UPLC HSS T3 column (1.8 um 2.1×100 mm). The mobile phases consisted of 0.1% formic acid in water (phase A) and 0.1% formic acid in acetonitrile (phase B), under both positive and negative ionization modes.

Raw data were acquired using MassLynxv4.2 and processed with Progenesis QI software for peak extraction, alignment, and normalization. Flavonoid quantification was based on total peak area normalization to determine the relative content across samples (Chen et al., 2013).

### 2.5 Weighted gene co-expression network analysis (WGCNA)

DETs and metabolite content data were used to construct co-expression networks using the WGCNA.v1.72 of R software (Langfelder and Horvath, 2008). TPM values of all AS transcripts were used for unsigned co-expression network analysis and soft -threshold calculation. The detailed parameters are as follows: power = 7, maxBlockSize = 3000, minimum module size = 100, and branch merge cut height = 0.45. After gene clustering, different modules were identified, and metabolite content are correlated with each module. Key modules showing correlation coefficients greater than 0.7 with specific metabolites were selected for further analysis.

### 2.6 Validation of Alternative Splicing Events

Total RNA was isolated from different tissues using the cetyltrimethylammonium bromide (CTAB) method. After evaluating the quality of the extracted RNA, first-strand cDNA was synthesized using the PrimeScript RT Reagent Kit (cat 6110A, Takara, Japan) according to the manufacturer’s instructions. Sequences of AS transcripts were obtained from the assembled GTF file using the *gffread* script in the Cufflinks software. Specific primers for these AS transcripts were designed using Primer5 and used for RT-PCR amplification. Details of all the relevant primers are listed in **Supplementary Table S1**. All amplification products were verified to be consistent with the reference sequences. Full-length and AS transcript structures were visualized using the Gene Structure Display Server (GSDS 2.0, https://gsds.gao-lab.org/) based on the GTF files. The expression levels of each transcript in different organs visualized as heat maps generated in Tbtools. Meanwhile, all bHLH gene family members were retrieved from the *Arabidopsis* database TAIR (https://www.arabidopsis.org/). The NJ phylogenetic tree was constructed using MEGA11 to verify the classification of *bHLH* genes in *Rosa roxburghii*, and their homologous relationships were further validated via NCBI.

### 2.7 Quantitative Real-Time PCR (qRT-PCR)

Expression patterns of selected AS transcripts across tissues were evaluated using qRT-PCR. The PrimeScript RT Reagent Kit (cat. RR036A, Takara, Japan) was used to synthesize first-strand cDNA according to the manufacturer’s protocols. *RrActin* was used as the internal control gene (Huang et al., 2019). Expression assays were conducted with three biological replicates, each with technical triplicates. The relative gene expression values were analyzed using the 2^-△Ct^ method.

## 3 Results

### 3.1 Landscape of AS in chestnut rose

Based on RNA-seq data from different tissues, we identified 8,586 genes in chestnut rose undergoing AS, resulting in 49,523 AS events. We conducted a statistical analysis of four major types of AS events and found that there were 13,574 AS events, IR was the most prevalent, with 13,574 events, accounting for 27.41% of the total AS events. This was followed by A3SS, A5SS, and ES, with 8,634 (17.43%), 6,445 (13.01%), and 4,376 (8.84%) events, respectively (**Table 1**; **Supplementary Figure S2**). In addition, numerous other AS types were detected, reflecting the presence of multiple splicing forms within single transcripts. For example, the Rr100087 gene produces the AS transcript MSTRG.65.1, which produces both ES and A5SS types (**Supplementary Figure S3**).

**Table 1:** Statistics of four major AS events obtained from RNA-seq in chestnut rose.

AS type	AS events number	Percentage
Intron retention (IR)	13,574	27.41%
Alternative 3’ splice sites (A3SS)	8634	17.43%
Alternative 5’ splice sites (A5SS)	6445	13.01%
Exon skipping (ES)	4376	8.84%
Others	16,494	33.31%
Total	49,523	100%

**Figure 1:**
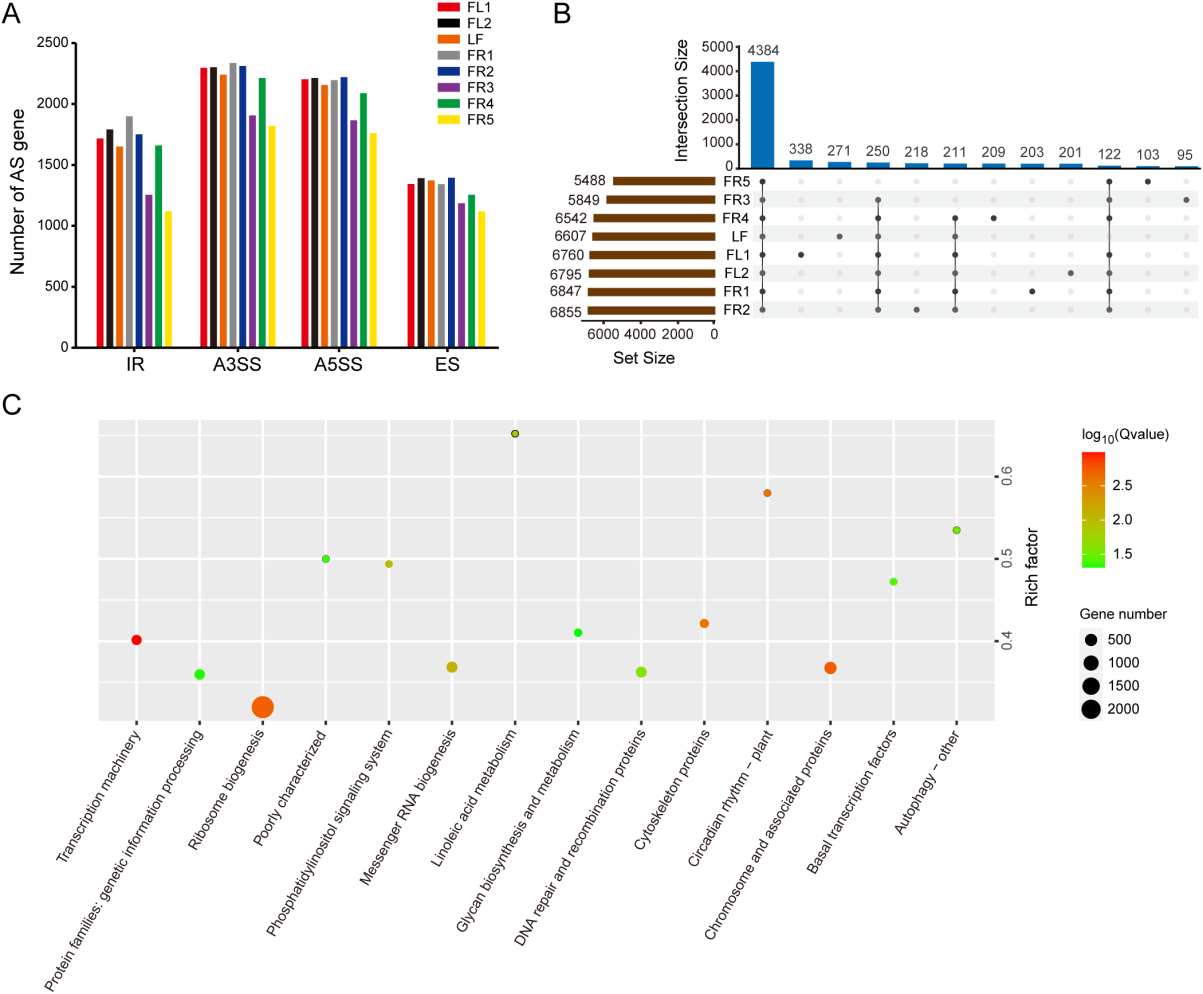
Analysis of AS genes in chestnut rose. **(A)** Statistics on the number of AS genes of four main types in eight samples. **(B)** Up-set venn diagram displays specific and conserved AS genes in eight samples. **(C)** KEGG enrichment analysis of differentially expressed AS genes with *P* value < 0.01. The color and size of the circles represent the p-values and gene number, respectively.

To investigate tissue-specific differences in AS, we conducted a statistical analysis of the number of AS gene counts across the eight sampled tissues. The results showed slight differences in the number of the four main types of AS genes occurring in different tissues (**Figure 1A**). Notably, the number of AS genes identified in FR3 and FR5 was lower than in other tissues. In addition, there were more AS genes with A3SS and A5SS types in all samples, followed by IR -type genes, with ES -type genes being the least common.

It is worth noting that although the number of AS genes involved in IR type is less than A3SS and A5SS, the number of AS events is the highest, indicating that one gene produces more IR transcripts. Venn diagram analysis showed that 4,384 (51.06%) of the 8,586 AS genes were conserved across all tissues, indicating a core set of AS genes shared among different tissue types (**Figure 1B**). We also identified numerous tissue-specific AS genes. For example, 271 AS genes were only identified in leaves. Similarly, 338, 201, 203, 218, 95, 209, and 103 tissue-specific AS genes were also identified in FL1, FL2, FR1, FR2, FR3, FR4, and FR5, respectively. Functional enrichment analysis of these tissue-specific AS genes revealed significant associations with pathways such as ribosome biogenesis, transcriptional machinery, and linoleic acid metabolism (**Figure 1C**).

### 3.2 Differences in flavonoid content between different tissues

Flavonoids are abundant in chestnut rose and are known for their diverse health benefits. To investigate their distribution, we used LC-MS to detect the flavonoid content in different tissues. A total of 45 flavonoid metabolites were detected across the eight tissue samples, with content levels varying significantly by tissue type.

**Figure 2:**
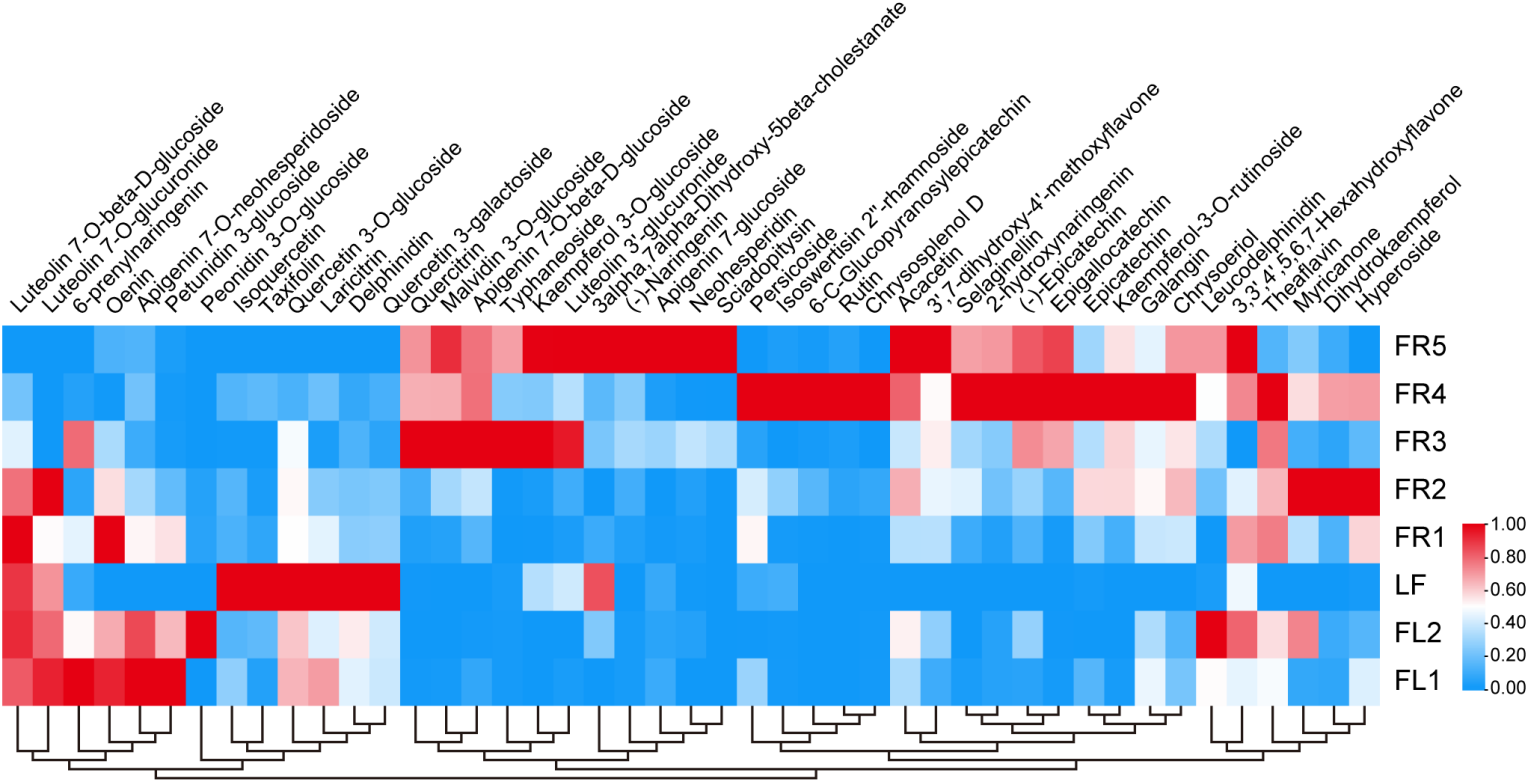
Analysis of differential flavonoid content in eight samples. Metabolite levels were clustered based on their average content. The blue and red colors in the heatmap represent low and high material content, respectively.

Delphinidin, taxifolin, and laricitrin were found at markedly higher levels in leaves compared to other tissues; notably, delphinidin content in leaves was more than five times higher than that in FR4 (**Figure 2**). Epicatechin, epigallocatechin, and dihydrokaempferol were most abundant in fruits, whereas petunidin 3-glucoside and peonidin 3-O-glucoside were more enriched in flowers.

In addition, we found that the accumulation of certain flavonoids corresponded with specific stages of tissue development. For example, petunidin 3-glucoside content in FR1 was higher than in FR2, and epigallocatechin levels in FR5 were more than tenfold higher than in FR1.

### 3.3 Co-expression analysis of AS genes related to flavonoid metabolism

**Figure 3:**
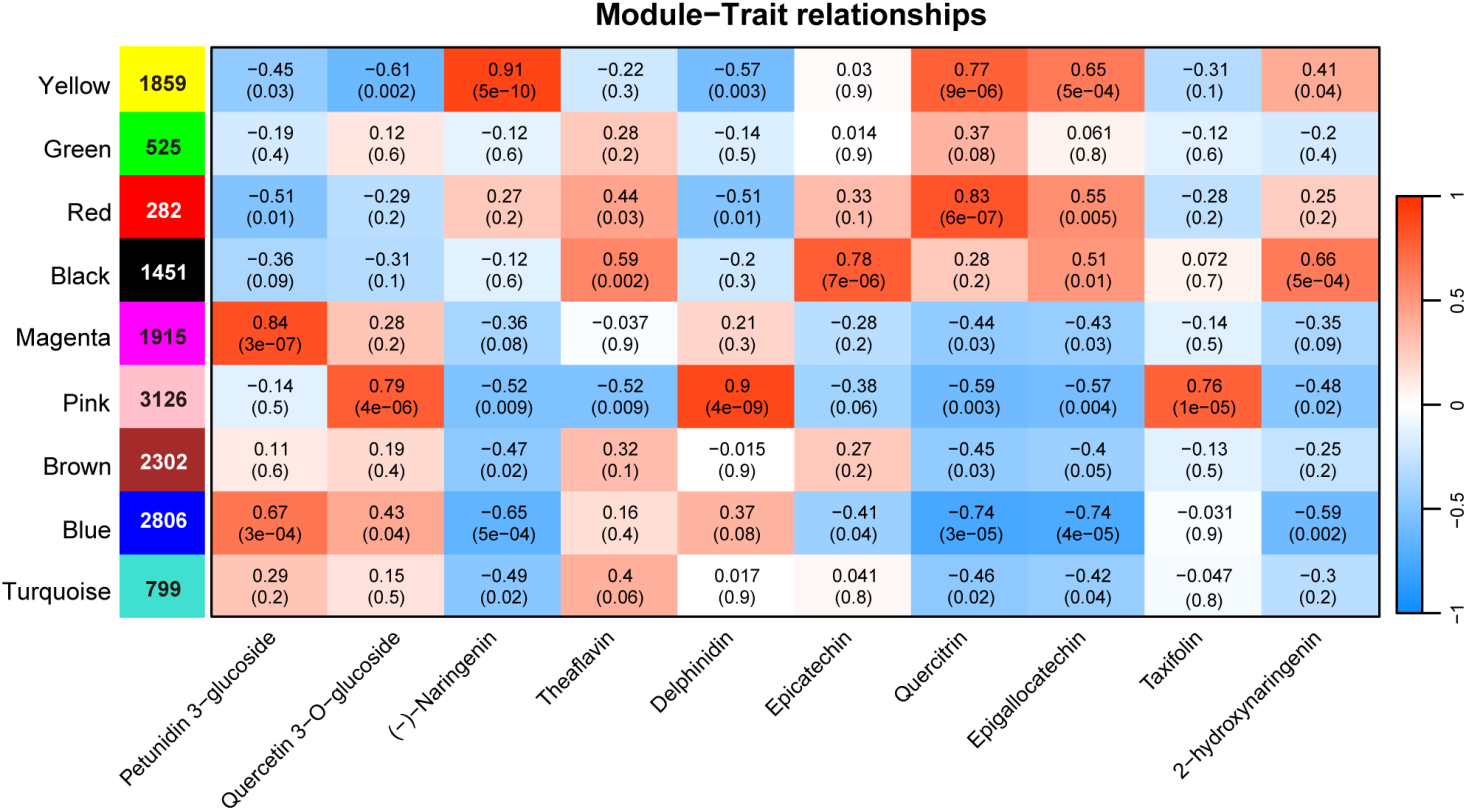
WGCNA of differentially expressed AS genes. The color blocks on the left represent the clustering of AS genes. The blue and red colors in the figure represent the negative and positive correlations between gene modules and metabolite content.

In order to investigate the relationship between flavonoid content and DETs, we performed weighted gene co-expression network analysis using transcript expression levels and the quantified content of 10 representative flavonoid compounds, including naringenin, delphinidin, and quercitrin, which showed significant variation across different tissues. The DETs were clustered into nine co-expression modules (**Supplementary Figure S4**). Of these, five modules—yellow, red, black, magenta, and pink—showed strong positive correlations with specific metabolites (r > 0.75, *P* < 0.001) (**Figure 3**). Specifically, the yellow, red, black, magenta, and pink modules contained 1,859, 282, 1,451, 1,915, and 3,126 DETs, respectively. The DETs in the pink module were significantly correlated with the contents of quercetin 3−O−glucoside, delphinidin, and taxifolin, with the strongest correlation coefficient (0.9) confirmed for delphinidin. DETs in the yellow module were correlated with naringenin content, with a correlation coefficient of 0.91. The contents of epicatechin, quercetin, and petunidin 3-glucoside were correlated with DETs in the black, red, and magenta modules, respectively. It is worth noting that, except for the DETs in the blue module that showed a negative correlation of -0.7 with quercetin and epigallocatechin, no other modules showed a significant negative correlation with these metabolites. The five modules we identified as significantly correlated with the content of these metabolites were also positively correlated, suggesting that the AS genes in these modules may be involved in the synthesis of these metabolites.

**Figure 4:**
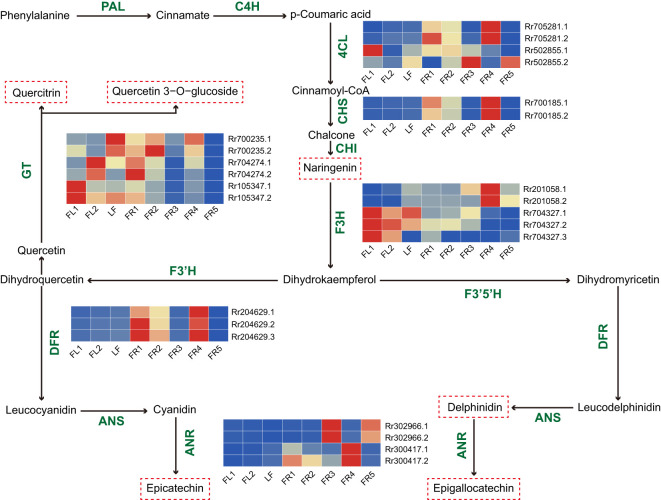
Flavonoid biosynthetic pathway and AS gene expression analysis. The red dashed box represents the differential flavonoid substances identified in the study. Heat map showing the relative expression level of AS genes with mean values of three biological replicates in eight samples.

The synthesis of plant flavonoids originates from the phenylpropanoid metabolic pathway, which is catalyzed by phenylalanine ammonia-lyase (*PAL*) and ultimately forms different flavonoids and their derivatives through the catalysis of multiple key enzymes. Through WGCNA, we identified key modules associated with the synthesis of these compounds. To further screen for key AS genes involved in these synthetic processes, we screened the modules for genes participating in known flavonoid synthesis pathways. A total of 11 AS genes and 24 transcripts were identified (**Figure 4**), including those encoding 4-coumarate coenzyme A ligase (*4CL*), chalcone synthase (*CHS*), flavanone 3-hydroxylase (*F3H*), dihydroflavanol 4-reductase (*DFR*), glycosyltransferases (*GT*), and anthocyanidin reductase (*ANR*). Among these genes, *F3H* (Rr201058) and DFR (Rr204629) produced three transcripts through AS. In addition, we analyzed the expression patterns of these AS transcripts across eight tissues using heat maps. The results showed that some AS transcripts had expression patterns consistent with their full-length transcripts, while others showed significant differences. For example, both F3H transcripts (Rr201058.1 and Rr201058.2) had the highest expression levels in FR4, while *ANR* transcripts (Rr302966.1 and Rr302966.2) showed elevated expression in FR3. Within the *4CL* and GT families, Rr502855.1 was highly expressed in flowers, while Rr502855.2 was more abundant in fruits. Similarly, Rr700235.1 exhibited peak expression in LF, while Rr700235.2 was highest in FR2. These results suggest that the functions of AS transcripts and their full-length transcripts may have synergistic effects or perform different functions.

### 3.4 Detection of AS in genes involved in the flavonoid pathway

**Figure 5:**
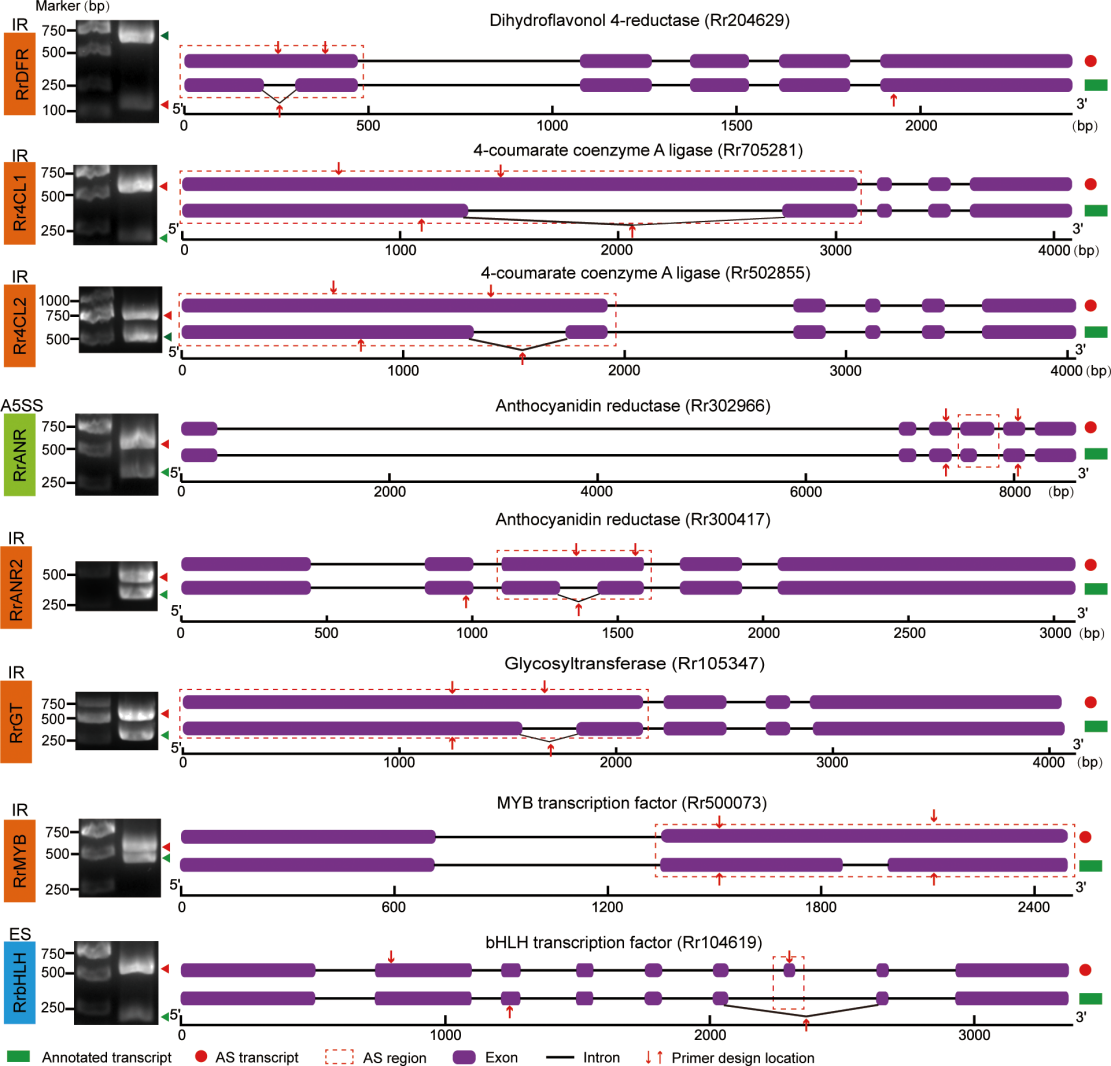
AS isoforms associated with flavonoid content. Agarose gel electrophoresis is shown on the left and green triangles represent AS and annotated transcripts respectively. The red dashed box represents the position where AS event occurs.

**Figure 6:**
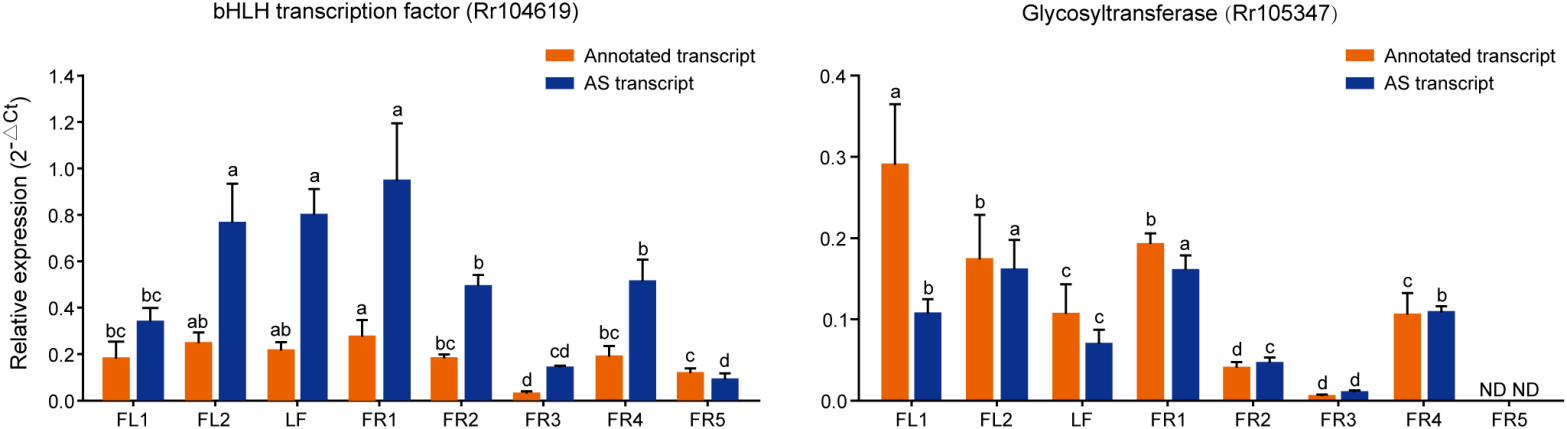
Expression of AS genes related to flavonoid content analyzed by qRT-PCR. 2^-△Ct^ is used to represent its relative expression level. The lowercase letters represent significant differences at the P < 0.05 level.

To verify the authenticity of AS events identified from the RNA-seq data, we designed specific primers in the common regions or regions flanking the AS sites of selected genes and performed RT-PCR and sequencing. Verification revealed that *DRF*, *4CL*, *ANR*, and *GT* genes underwent AS to produce two different transcripts (**Figure 5**). Additionally, *MYB* and *bHLH* transcription factors, known to regulate flavonoid synthesis in plants, were identified in five key modules of the WGCNA. The corresponding GO enrichment results are shown in **Supplementary Table S2**. After filtering for low-expression genes and analyzing their correlation with metabolites, we identified one *MYB* and one *bHLH* gene in the yellow module (**Supplementary Figure S5**). Both transcription factors have been widely reported as key regulators of flavonoid synthesis and were confirmed here to undergo AS events (**Figure 5**). All gel images from the specific PCR amplification of AS genes are shown in **Figure 6**. For instance, primers designed in the common region of *MYB* full-length and IR transcripts amplified two distinct bands of approximately 600 bp and 500 bp, respectively.

Among the eight validated AS genes, RrDFR, *Rr4CL1*, *Rr4CL2*, *RrANR2*, *RrGT*, and *RrMYB* all exhibited IR-type AS events; *RrbHLH* underwent ES-type AS events; and *RrANR* showed A5SS-type AS events. Many studies have shown that IR can lead to premature termination of translation, resulting in truncated proteins. For instance, the Rr4CL1 gene produces a truncated protein after an IR event—its full-length transcript encodes a 587-amino-acid protein, whereas its AS transcript encodes a 403 -amino-acid protein. Interestingly, agarose gel electrophoresis showed that the expression level of the AS transcript of *bHLH* was significantly higher than that of its annotated transcript, suggesting that the AS transcript may function as a major isoform. Additionally, the ES event led to a 15-amino-acid difference between the two transcripts, although this variation did not occur within the functional domain. Homologous alignment using NCBI and phylogenetic analysis showed that this *bHLH* gene is most closely related to AT4G29100.1 (*AtbHLH68*) in *Arabidopsis thaliana* (**Supplementary Figure S6**). To separate the two bands in agarose gel electrophoresis, we designed specific primers to amplify the bHLH transcripts individually, creating a size difference of approximately 250 bp (**Supplementary Table S3**).

### 3.5 Expression patterns of key AS genes in different samples

In order to further explore the function of these AS transcripts in the growth and development of chestnut rose, qRT-PCR was used to analyze their expression patterns across different tissues. The results showed that the expression level of the *bHLH* AS transcript was significantly higher than that of its annotated transcript, indicating that the AS transcript may exist as a major isoform. Notably, the AS transcript showed high expression in FL2, LF, and FR1, but relatively low expression in FR3 and FR5, indicating that it may play different roles in tissue development and fruit maturation. While the expression trends of the annotated and AS transcripts were generally similar, both also showed high expression in FL1 (**Figure 6**). The annotated transcript of the *RrGT* gene showed the highest expression in FL1, followed by FL2 and FR1, with lower levels in FR3 and no expression in FR5. Its AS transcript was most highly expressed in FL2 and FR1, suggesting that the two transcripts may function differently at various stages of flower development (**Figure 6**).

Correlation analysis with flavonoid content revealed that both the annotated and AS transcripts of *bHLH* were significantly positively correlated with delphinidin, indicating a potential regulatory role in its synthesis. The GT gene, a glycosyltransferase, also showed a significant positive correlation with both petunidin 3-glucoside and quercetin 3−O−glucoside (**Supplementary Figure S5**).

## 4 Discussion

As an important post-transcriptional regulatory mechanism, AS is widespread in plants. For example, it has been found that 70% of genes in *Arabidopsis* can undergo AS, with IR being the main type of AS event (Martín et al., 2021). Similarly, construction of the tea plant AS database revealed that about 56.7% of genes in tea plants can undergo AS (Mi et al., 2021). However, the characteristics of AS in chestnut rose were previously unclear. In this study, among 40,020 annotated genes, 8,586 were found to undergo AS, accounting for 21.45% of the total, with IR as the most common AS type. Compared with other *Rosaceae* species, 57.67% of genes in strawberries (Li et al., 2017) and 40.15% in apples can undergo AS, with IR being the predominant type in both cases (Zhou et al., 2022). This indicates that there are differences in the number of AS events between different species, but IR consistently appears as the most common type. Organ-specific AS analysis showed that 4,384 (51.10%) AS genes were conserved and exhibited AS events across all eight tissues. In contrast, a smaller number of genes showed tissue specificity, such as the 271 AS genes identified only in leaves. Similar tissue-specific AS events have also been found in tea plants (Zhu et al., 2018). Our results provide insights into the basic overview and characteristics of AS in chestnut rose.

In addition, previous studies have found that AS can help regulate plant phosphorus signaling and growth (Guo et al., 2022). In our study, we identified tissue-specific AS events and differentially expressed AS genes, which may contribute to the spatial regulation of chestnut rose development. However, further research is needed to identify and analyze the functions of these key genes.

AS plays an important role in plant growth, development, and resistance to biotic and abiotic stresses (Cheng et al., 2024; Alhabsi et al., 2025). Although chestnut rose is rich in flavonoids, the role of AS in flavonoid synthesis remains unclear. By identifying AS events across different tissues, quantifying flavonoid content, analyzing differentially expressed AS genes, and conducting WGCNA, we focused on AS genes potentially involved in flavonoid synthesis, including *4CL*, *ANR*, *DFR*, *MYB*, and *bHLH*. For instance, AS of the *DFR* gene in eggplant has been linked to anthocyanin accumulation in petals and peel (Wang et al., 2022). Similarly, the expression of *4CL*, *ANR*, and *DFR* AS genes is significantly correlated with flavonoid content, supporting the relevance of our findings (Zhu et al., 2018). The study of these AS genes is similar to the genes we discovered, demonstrating that these AS may play a role in the synthesis of flavonoids. Certain flavonoids can generate corresponding glycosides through the action of glycosyltransferases (Dai et al., 2022). In citrus, *CitUGT72AZ4* can specifically catalyze the glycosylation reaction of flavonoid compound 4’-hydroxyl, as well as the generation of glycosides from quercetin (Liao et al., 2025). We found that three glycosyltransferase genes can induce AS and are associated with flavonoid content, suggesting a possible post-transcriptional regulatory mechanism for flavonoid glycoside formation. Notably, the two AS transcripts of Rr105347, a glycosyltransferase, displayed distinct tissue-specific expression patterns: the annotated transcript was most highly expressed in FL1, while the AS transcript was predominant in FR1 and FL2, indicating potential differences in their biological functions.

As an important transcription factor, *bHLH* has been confirmed to regulate the synthesis of flavonoids in multiple plants. For example, the bHLH1–DTX35/DFR module has been shown to regulate flavonoid synthesis in chili peppers (Zhang et al., 2022). In addition, studies have found that *CsbHLH1* undergoes AS events that lead to the loss of structural domains, resulting in the absence of anthocyanins in white cornflower (Cheng et al., 2024). In this study, the AS gene *bHLH* was found to be associated with flavonoids. This gene produces two transcripts, and except in FR5, the expression level of its AS transcript is significantly higher than that of its annotated transcript, suggesting that the AS transcript functions as the main transcript in chestnut rose. This suggests that AS transcripts may have more important functions compared to annotated transcripts. Different transcripts may have the same or opposite functions, thereby playing a synergistic or antagonistic role in regulating specific traits (Wu et al., 2019; Li et al., 2021). The expression patterns of the two transcripts are similar: both are highly expressed in FL2, LF, and FR1, and show relatively low expression in FR3 and FR5. The ES event identified in this gene does not occur within a critical structural domain, suggesting its function may remain unchanged. Although many studies have shown that *bHLH68* contributes to stress resistance, its role in flavonoid synthesis remains unclear. Some studies suggest that its homologs may promote the accumulation in plants, which could further regulate flavonoid metabolism (Liu et al., 2017; Fang et al., 2025). Moreover, studies in *Arabidopsis thaliana* also show that the interaction between *bHLH68* and *MYB48* may be involved in the regulation of the flavonoid synthetic pathway (Guo, 2022).

## 5 Conclusion

In this study, the AS of chestnut rose was investigated using transcriptome sequencing data. Our analysis focused on the flavonoid synthesis pathway and confirmed that AS plays an important regulatory role in this process. These findings provide insights into the basic characteristics of AS and tissue-specific AS events in chestnut rose and elucidate the new mechanism underlying flavonoid synthesis in this species.

## Data Availability

The datasets presented in this study can be found in online repositories. The names of the repository/repositories and accession number(s) can be found in the article/**Supplementary Material**.
