# Supplementary figures and images for: Comprehensive analysis of alternative splicing in Rosa roxburghii Tratt reveals its role in flavonoid synthesis
Source: Front Plant Sci. 2025 Jul 11;16:1627126. doi: 10.3389/fpls.2025.1627126 (PMC12291298; doi:10.3389/fpls.2025.1627126)

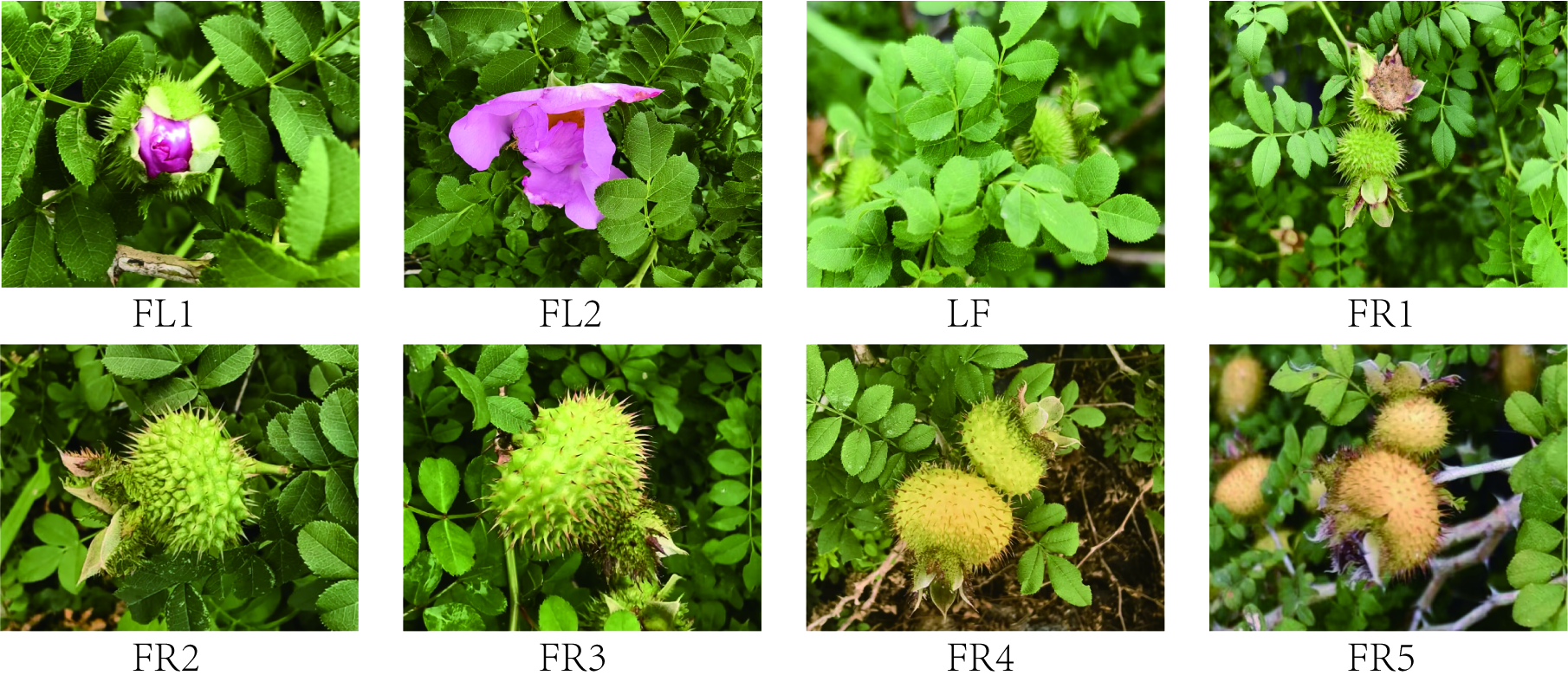

Supplement: Supplementary file 1 [file Image1.tif]

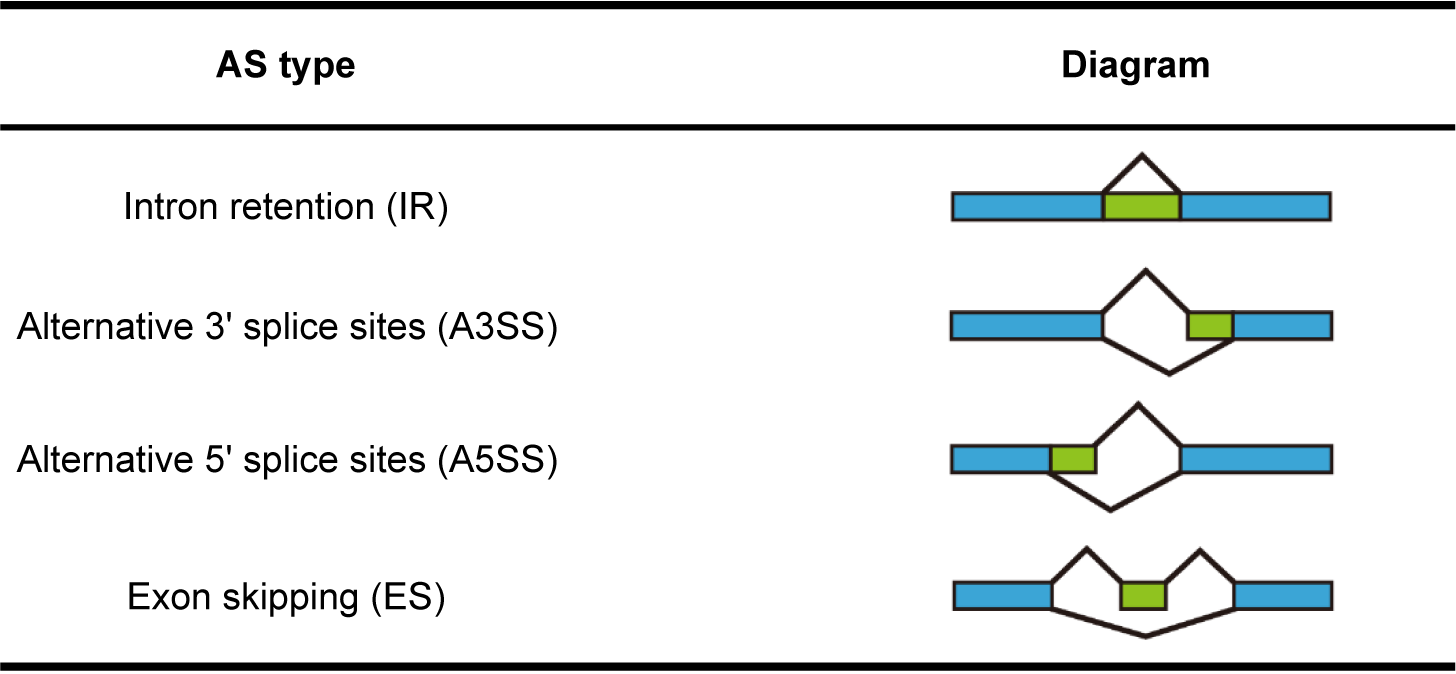

Supplement: Supplementary file 2 [file Image2.tif]

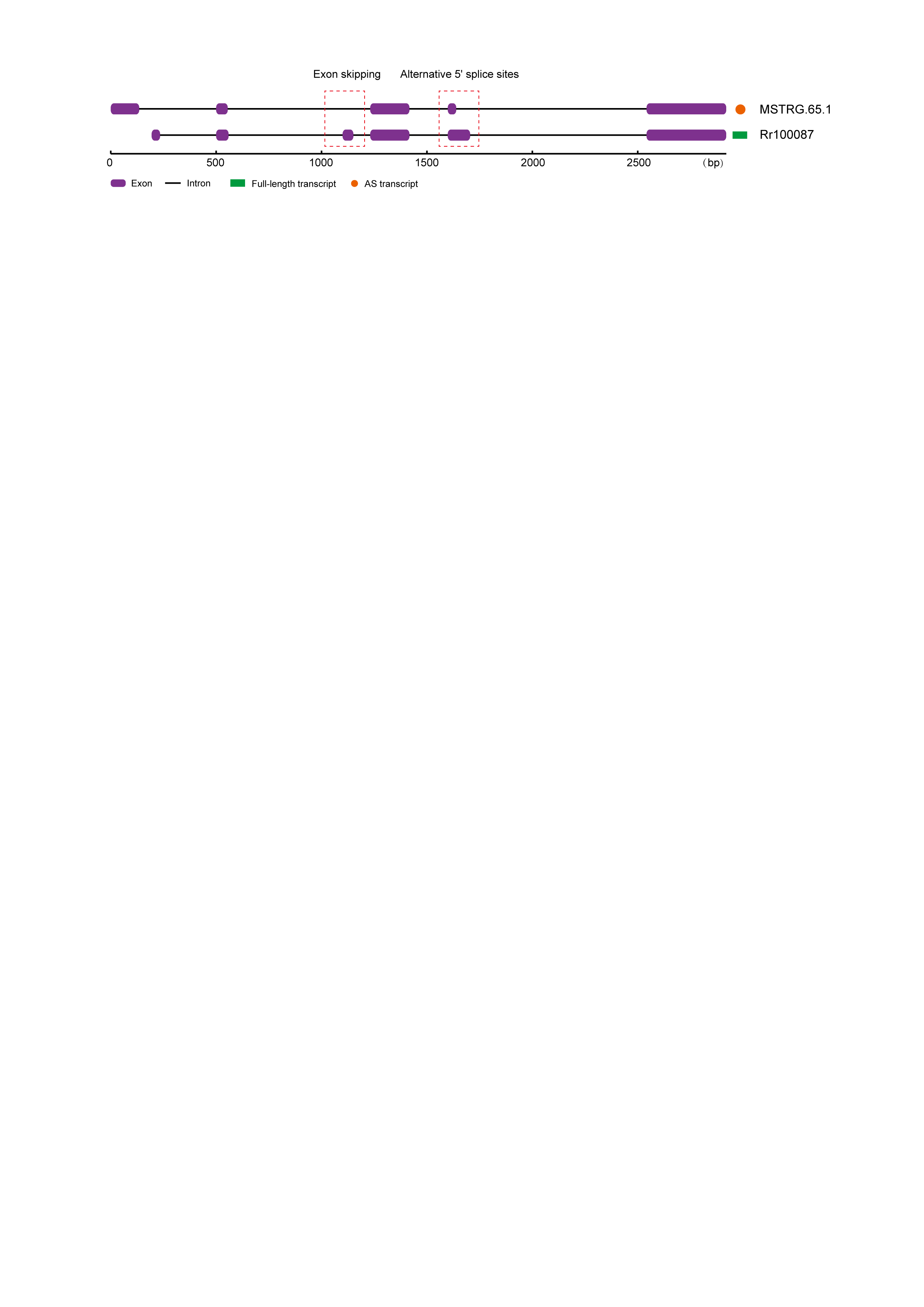

Supplement: Supplementary file 3 [file Image3.tif]

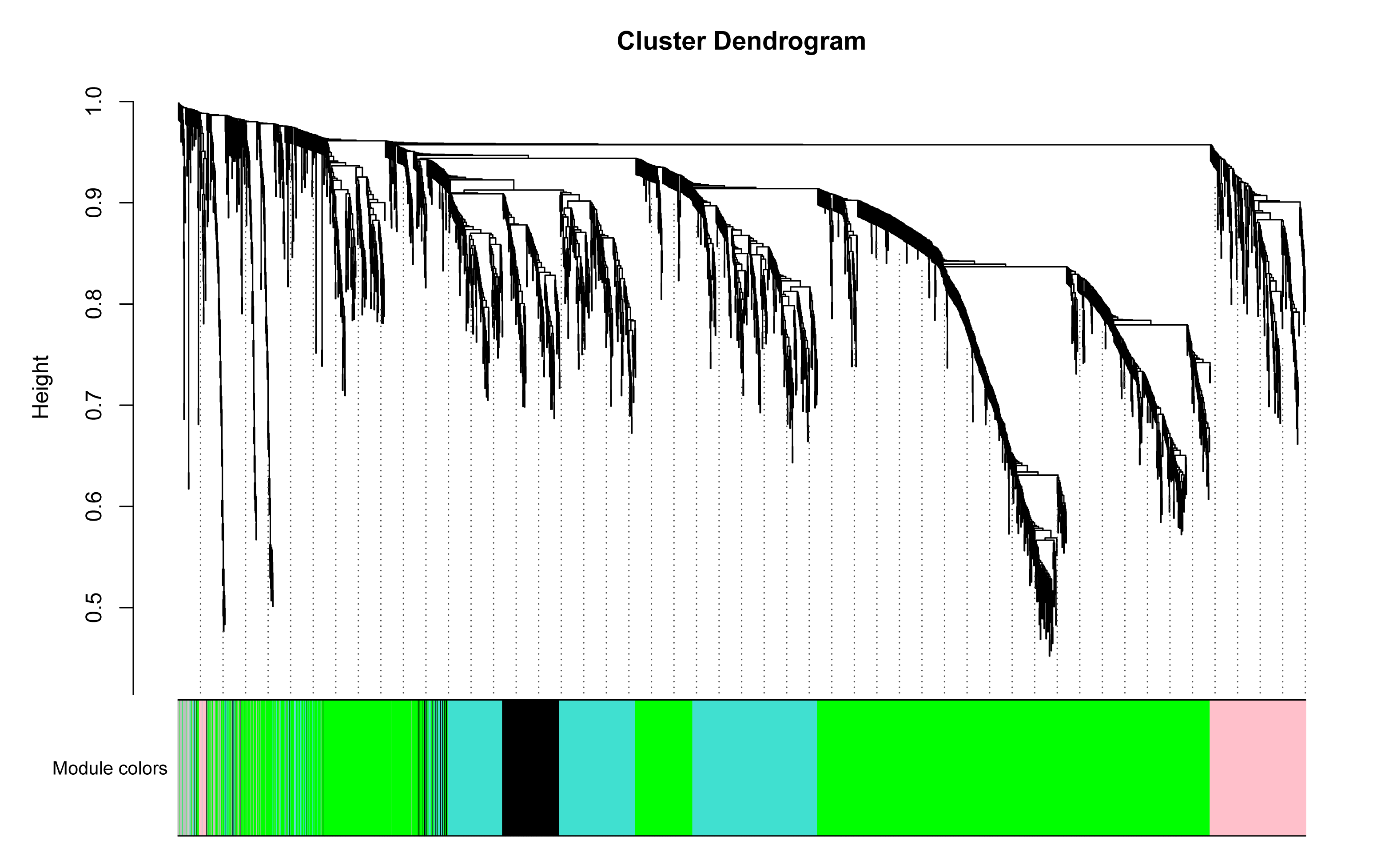

Supplement: Supplementary file 4 [file Image4.tif]

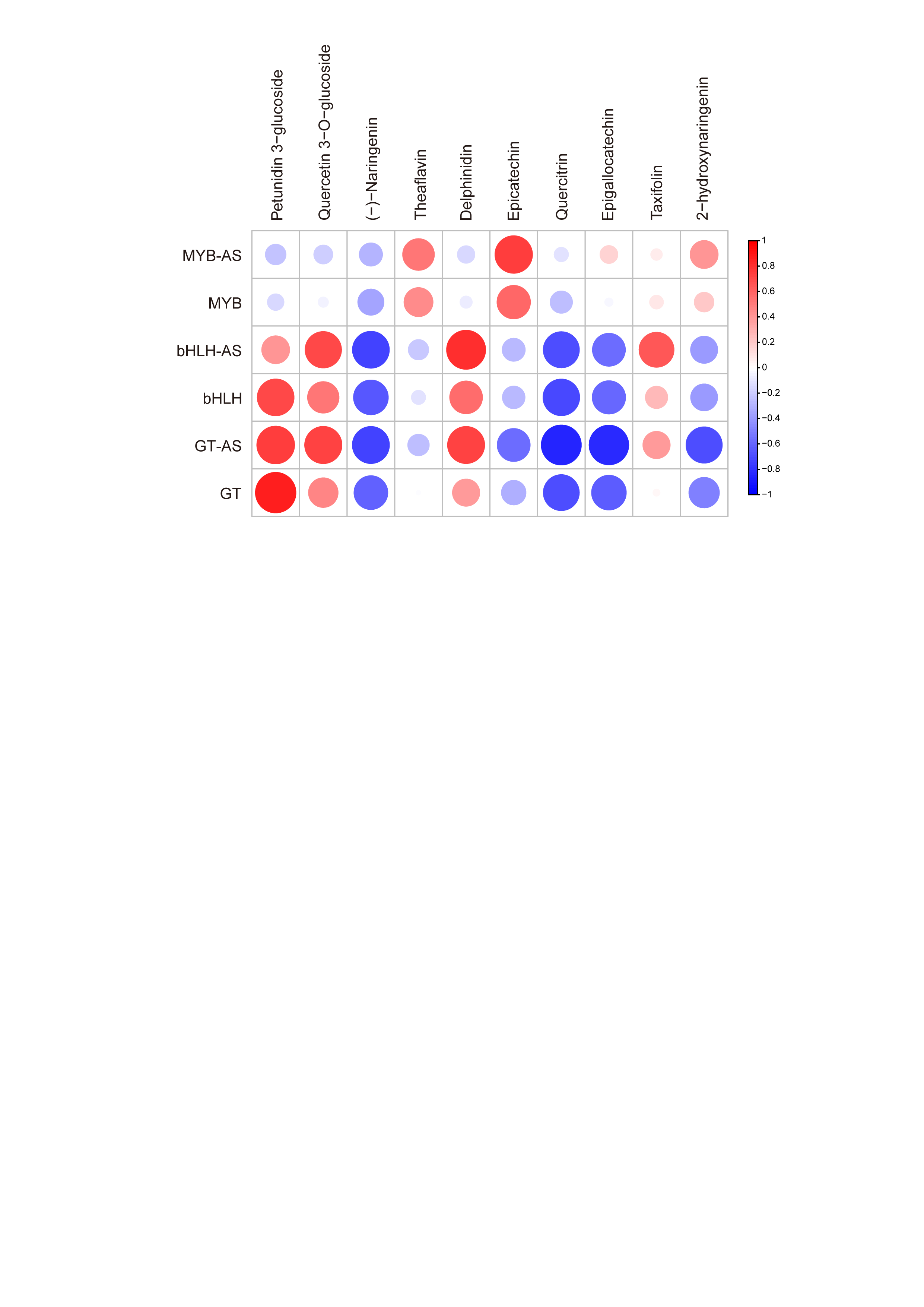

Supplement: Supplementary file 5 [file Image5.tif]

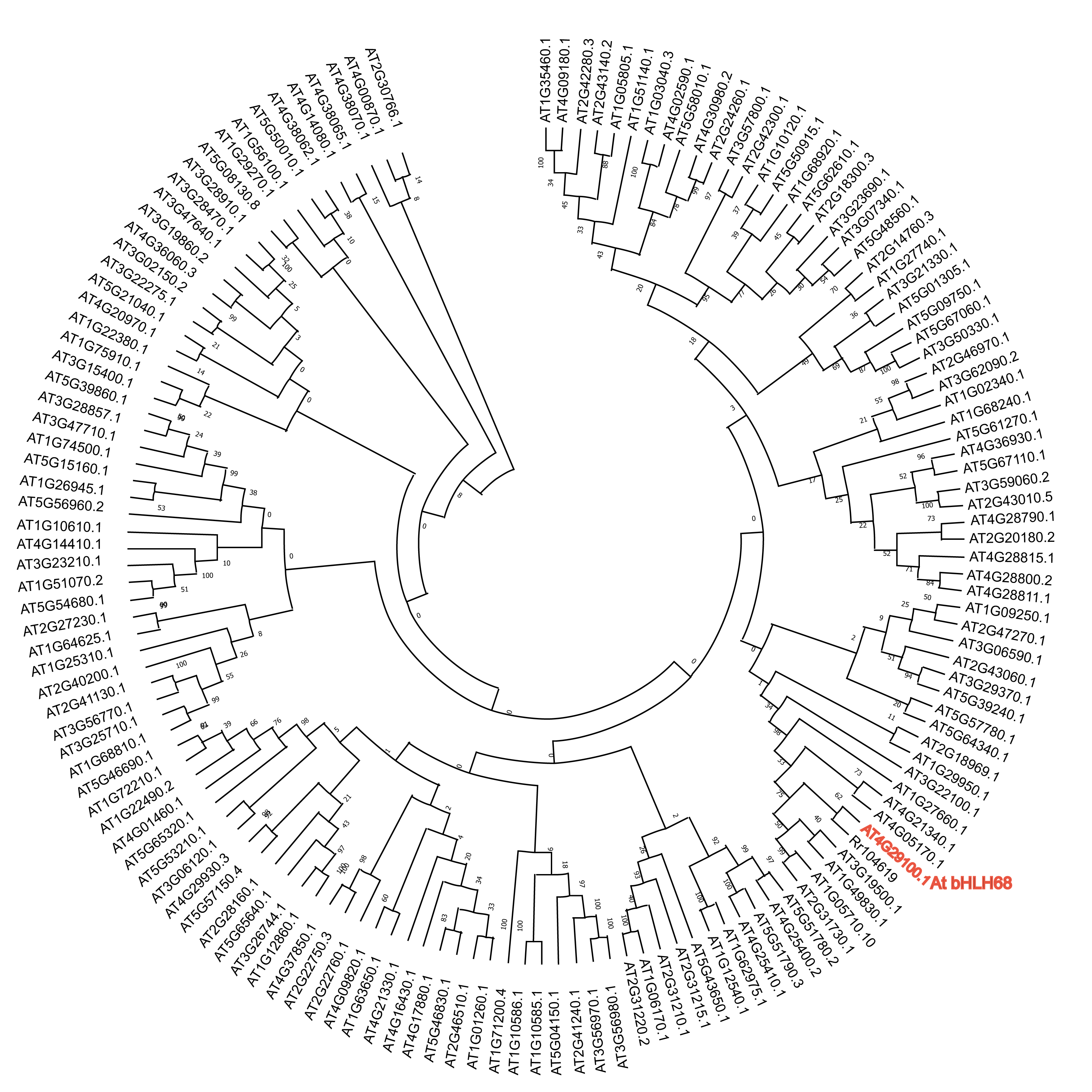

Supplement: Supplementary file 6 [file Image6.tif]
