# Supplementary material for: Comprehensive analysis of alternative splicing in Rosa roxburghii Tratt reveals its role in flavonoid synthesis
Source: Front Plant Sci. 2025 Jul 11;16:1627126. doi: 10.3389/fpls.2025.1627126 (PMC12291298; doi:10.3389/fpls.2025.1627126)
Supplement: Supplementary file 9 [file Table3.docx]

> bHLH-AS gene

TTGAAAGTTTGAAACAAGAGAAACACATAAGCAAAAGGTTTCTCAATCCACCCTCTCTATAAGTTTTCTTTAAGGCCACAGACTTATAAGCTTCCAATTCAACCTCATCGGAGAAAAGCAAATAGTACCCACAAAAGCTTTTAGCTAGCAAAGTCTTGTTTGGGTGCACATACAGGGAAATATAGAAAAGTGAGAAAGAAAGCAAAAGAGAAAGGAAAGCAAAGGGAACCAAAGAAGAGGAAAGGAGAATTCTTTTTTAGTTTCCTTTGTGATCTTTTGATCGTTCAAAGCGCGCATGAAGTAGTGAGCAGAAGACGCTAGTGTTTTTCATTAGTGATCATAAGTGCAACAAATGATGGCCGGAAACCCTAACTGGTGGAGCACGCATCCACCGTCATCTATCTTCCCTTCTCAATATGTGCTTGGATCTTCTAATTCACTCCCTCAGCTCAATTCCTTCCCTGATCAGCATAACCTACAGCCTCCTCAGTCTTGGAGCCAATTACTTCTGGGCGGATTGTCCGGAGAAGATCAAGACAACTTTGGAAGTTTGAATCATTTTCCACCTAAAAGGTTGGAAAGTTGGGAGGATCATCAGCAGCAAATATTGATTCCACAGTCGTATCCAAGGATTAATCCTGTTGTTGATAGTAATATTATAAAGCAAGAGGTTAATTACCAGAACAACAGCAACTTGTGTGGTGGAGATGGAGGAGAAGAATTTCAGGCACCTTATAATAATAGGCCATCTTGGTCAGCAGCACAAATCAACATGCCAGTTTCATCCCCTAGGTCTTGTATCACTAGTTTAAGTAGTAATAATATATTGGACTTTTCCTATAACCAGAAGAACCAACATGCAGATCACTCATCTGAGTGCAATAGTCCTCCCATCGGCGGAATGTGCAAGAAAGCTAGGGCTCAACCATCTTCAAGCCAACCACCATTAAAGGTGAGAAAGGAGAAGCTAGGAGATAGAATAACAGCCCTTCATCAAATAGTTTCCCCATTTGGAAAGACTGACACTGCTTCTGTCTTGTTAGAAGCCATTGGGTATATCAGATTCCTCCAGAGTCAAATTGAGGCCCTCAGCTCCCCTTACTTGGGCAATGCCTCCAAAAACATGAGGAATAATCCCCACTCTGTTCAAGGAGAAAGGAATTGTGTGTTTCCTGAAGACCCTGGTCAGCTGTTGAATGACAACTGCAGCCTGAAAAGAAAAGGGGCTTCTAACCAGGATTCTCATCAAGACTATAAGGCAAAGGATTTAAGAAGTAAAGGATTGTGTTTGGTTCCTGTTTCTTGCACTCAGAATGTTGGGGGTGACATTAACATTGGAGCTGATTATTGGGCTCCGGCTTATGGGAGCGGGTTTTAGTTTGAGACTCTAGGACTAGATGCAGCGCGTATATTTGTATGGTACGCTGCTTTTGTAGAATGAGATAACATAATGAGCATTCACTCTGTTAGAATTTAATAATTAAGATTGATTGCTCATCATGCTATTTGCATTCTAAATTAGAAGTGTACTGTTGCGAGCTAGTAATTTATTGCACTACTTAACTAATTAATTAAATAAATTAAGCTACTCTCTCTCTCTCTGTCAAGATCTTGCAAGCTTAATTATTCTAAGTAATATAATTTTTTTTAATATAAACTCAATTTGG

>bHLH-annotated gene (Rr104619)

TTGAAAGTTTGAAACAAGAGAAACACATAAGCAAAAGGTTTCTCAATCCACCCTCTCTATAAGTTTTCTTTAAGGCCACAGACTTATAAGCTTCCAATTCAACCTCATCGGAGAAAAGCAAATAGTACCCACAAAAGCTTTTAGCTAGCAAAGTCTTGTTTGGGTGCACATACAGGGAAATATAGAAAAGTGAGAAAGAAAGCAAAAGAGAAAGGAAAGCAAAGGGAACCAAAGAAGAGGAAAGGAGAATTCTTTTTTAGTTTCCTTTGTGATCTTTTGATCGTTCAAAGCGCGCATGAAGTAGTGAGCAGAAGACGCTAGTGTTTTTCATTAGTGATCATAAGTGCAACAAATGATGGCCGGAAACCCTAACTGGTGGAGCACGCATCCACCGTCATCTATCTTCCCTTCTCAATATGTGCTTGGATCTTCTAATTCACTCCCTCAGCTCAATTCCTTCCCTGATCAGCATAACCTACAGCCTCCTCAGTCTTGGAGCCAATTACTTCTGGGCGGATTGTCCGGAGAAGATCAAGACAACTTTGGAAGTTTGAATCATTTTCCACCTAAAAGGTTGGAAAGTTGGGAGGATCATCAGCAGCAAATATTGATTCCACAGTCGTATCCAAGGATTAATCCTGTTGTTGATAGTAATATTATAAAGCAAGAGGTTAATTACCAGAACAACAGCAACTTGTGTGGTGGAGATGGAGGAGAAGAATTTCAGGCACCTTATAATAATAGGCCATCTTGGTCAGCAGCACAAATCAACATGCCAGTTTCATCCCCTAGGTCTTGTATCACTAGTTTAAGTAGTAATAATATATTGGACTTTTCCTATAACCAGAAGAACCAACATGCAGATCACTCATCTGAGTGCAATAGTCCTCCCATCGGCGGAATGTGCAAGAAAGCTAGGGCTCAACCATCTTCAAGCCAACCACCATTAAAGGTGAGAAAGGAGAAGCTAGGAGATAGAATAACAGCCCTTCATCAAATAGTTTCCCCATTTGGAAAGACTGACACTGCTTCTGTCTTGTTAGAAGCCATTGGGTATATCAGATTCCTCCAGAGTCAAATTGAGGCCCTCAGCTCCCCTTACTTGGGCAATGCCTCCAAAAACATGAGGAATAATCCCCACTCTCTGTTGAATGACAACTGCAGCCTGAAAAGAAAAGGGGCTTCTAACCAGGATTCTCATCAAGACTATAAGGCAAAGGATTTAAGAAGTAAAGGATTGTGTTTGGTTCCTGTTTCTTGCACTCAGAATGTTGGGGGTGACATTAACATTGGAGCTGATTATTGGGCTCCGGCTTATGGGAGCGGGTTTTAG

>GT-AS gene

TTCCTTTCTATCCATTTCCTTTGTCCTTGTTTATCAATACTCTAATCAAGTTCAATTACTTTAATCATACAGTAAGTTCCTCAATTTCTGCAACTTAATGCAAAATCCAAAACCCAATACCAAAGTGTTCTCCTTTTTTAAAAAAAAAGACTGCAGTATTCTAGCTCTTCCATTCTCACATGGTCTGCTACTTCATCTTAGTCAGAGCTTTAGTTGTTAATGGCTCATTGTAAGCTTTATCTTCACTTTCCCTAGTGATATAATATTTGTTCTTGATTGTACAGTTTCTTTGTAATGGATTTGTCTGTTTAAAAACTATTTTATTCTATTCTAGCAAATGGTTATATGTGGAAAGGTGATATGGGGGTTTGAGTGTGTTTCTTAAAAAGCTAATAAGATTGCTAATCATACCCCACACAGATTAAACGTTTAACTAATGGTGATTGACATTTTCTCTATAATGGTGATTTTCCATGTTGGGTAGCTATTGGAGCTTTTTGGTACTGTCAGAGAAACATCCACACACTCTTGCTTTCCTTCTCCTCTTCCTCTGCCTTTCTTAGTTTGCTGCATTTGGGTTTCAAATGCTGAGACTTGAACTGGTTTCGGTCAAGTCTTTTCAGTGTTGGATCTGATTCTTTGTACTAGGCCTCAATTGTGTTTGTCACTTTTCTCATCTTGGTTTCTGGGTTTTAAGGTTTCCTGCTATGTTACTGTTGTTTAGTGGTTTTAGACCTGAAATGGGGTTTTAGAATCTCAATCCTTGTAGATTTCTCTGTCATATTGAGGAATTTCAATCCTTTTGGTTCTGTACTTTATTTTAGTTCAGTTGAGAGGTCAAACATGGCTCCAAGTTTAGATTTTTCTAGGTGGTGGGGAAAGGACAACAATTCAAGTAAGGGTACTCCAGTGGTGGTGACTATGGAGAACCCAAATTACTCTGTACTTGAGATAAATGGTCCTGATGAAGTGTTCAGGCCAGTTGATAAGGACAGAGGCAAGAATGCCAAGCAGTTTACATGGGTTTTGCTTCTCAAGGCCCATAAAGCTGTTGGCTGTGTTGCTTGGCTTGGAAATGTTCTCTGGGACTTGCTTGGAGCCATTAAGAAAAGATTGGTTTTTGGGCAAATGGAGAATGAGAAATCTGGCAAAGGAATCTTGTATAGGGTCATTATGGGGTTCTTAGTGATGGCTTTGGCTTTTCTGGCTTTTGAAGTGGTTGCTCACTTGAAAGGTTGGCATTATTTTGAGAACAGCAGCTTACATATCCCTGAGACTCTGGAGATTAGGGGGTGGCTTCACTTGGTTTATGTGGCATGGTTGGAGTTCAGAGCTGACTGCATTGCACCTGCAATTCAAGCTTTGACGAACTTCTGTATTGCTCTCTTCTTAATCCAATCCGCAGACCGTATGCTGCTTTGTTTAGGTTGCTTCTGGATCAAGTTCAAGAAGATTAAGCCAAGAATTGAAGAAGTTCCATACAAGCCAGACGATTTGGAAGGTTCAGGTCGTAATTACCCCAAGGTTCTTGTTCAAATTCCAATGTGTAATGAGAGAGAGGTAAGTTTCTCAAGTCTTAATCTCTTTATTTTCTGAATTATGTAGGTTCTATAGGCGAATCTAATGTAAGAATAGCTAAATTTGATATAACTTTTGTTTTAAGCTTACTCACTATCCAAATTGAAATAACCTTATAAAAGGGAAAAGTAGCTAAATAAGTATAACAGAACAAATTGATGTTTTAGGAACTTTCTATTTTCCATAGATTTTTCATCATGTTGATGACAGTGACTTTTTATCATAAAACTGTCTTTAGGTATATGAACAATCTATTTCAGCAGTATGCCAACTTGATTGGCCTAAAGAACGCCTTCTGATTCAAGTTCTAGATGATTCTGATGATGAGAGCATACAGTGGTTAATTAAGGCAGAGGTAACTCAGTGGAGCCAAAAAGGTGTCAATATAATCTACCGGCATCGTTTGGTTAGAACTGGTTATAAAGCTGGGAATCTCAAGTCTGCAATGAATTGTGATTATGTGAAGGACTATGAGTTTGTTGCAATCTTTGATGCTGATTTCCAACCAAACCCTGACTTCCTCAAGCTGACAGTTCCCCATTTTAAGGACAATCCTGAGCTTGGGTTGGTTCAGGCGAGGTGGTCTTTTGTTAACAAGGATGAAAACTTGTTGACACGCCTCCAAAATATCAATTTGTGCTTCCACTTTGAAGTGGAACAGCAGGTTAATGGGGTTTTCCTCAATTTCTTTGGCTTCAATGGTACTGCTGGAGTTTGGAGAATTAAAGCACTTGAAGACTCTGGCGGTTGGCTTGAGCGTACAACAGTAGAAGACATGGATATAGCAGTTCGCGCTCATCTTAATGGGTGGAAGTTCATATTCCTTAATGATGTCAAGGTCCTCTGTGAACTTCCCGAGTCTTACGAAGCTTATAAAAAACAGCAACACCGTTGGCATTCTGGTCCGATGCATCTCTTCCGTTTGTGCCTCCCTGCAATAATTACTTCAAAGATGATGTTCTGGAAAAAGGCAAACTTGATACTACTATTCTTTCTCCTTAGGAAGTTAATCCTCCCATTTTACTCTTTCACATTGTTTTGCATAATACTTCCTTTGACCATGTTTGTCCCCGAAGCTGAGCTATCCATGTGGGTTATATGCTACGTGCCCGTTTTTATGTCATTCATGAACATTCTTCCTTCTCCTAGATCTTTTCCCTTCATTGTCCCCTACCTCCTGTTTGAAAACACAATGTCTGTGACCAAATTCAATGCCATGGTATCTGGGTTGTTCCAGTTGGGGAGCTCATATGAATGGATTGTAACCAAGAAGGCGGGACGATCATCAGAATCTGATCTGTTAGCTGTGGAGGAGAGGGAAACGAAGGCCATGAACCACCCACAACTTCATAGAGGAACTTCAGACAGTGGTCTCTCTGAGCTCAACAAGTTAAAAGAGCATCAAGAAGCTGCTGCTCCTAAACCTCCTGTAAAGAAATTGAACAAGATCTACAAGAAAGAGCTGGCACTCGCTTTCTTACTGCTCACTGCTGCAGGTAGAAGCCTTCTATCAGCTCAAGGGGTACATTTTTACTTCCTACTATTTCAAGGCATATCATTTCTACTGGTCGGTCTTGACCTGATTGGTGAGCAGATGAGCTAGTAATTATTGGCGTGAAAAGTTAATGGTAAAGATAGAAATAAAGGGTGAATAAGAGATGGCAAACCAATGTGGTTTTCAGCTTGGTGCACAAATTGGCACACAGGTAGAGATGCAAAGAGCTTCTTCTCATTAGAAGTGCCCATATAGATTCTTTGTCCTCGTATTGTTTGCCCATATAGATTCTTTGTCCTCGTATTGTTTGCCCATAACCTCACACCATCTTCTTGCTTCTCTGTTTTGGTTCGTAAATTATAGATTTCTGTATGGTTGGTATTCTTAGATGAGCCACAATGTATTCCAATACCAGATAGGTTGATTTCTGTAGGGAAGTGTTGGTTTGCATTTCAACATTTACAAATGTAAAACTTTCTGTATATTGCACCAGTTCATTTTATAACAAGATTATATTTCTGTCTTGTATCAAAAAAAGATTTTATTTCTGTCATTTTTCTTATTTGTGTAATTGTAAGGAAGTTTTATCACTATGAAATAAGAAATTGTGAAAAAGTGTC

>GT-annotated gene Rr105347

TTCCTTTCTATCCATTTCCTTTGTCCTTGTTTATCAATACTCTAATCAAGTTCAATTACTTTAATCATACAGTAAGTTCCTCAATTTCTGCAACTTAATGCAAAATCCAAAACCCAATACCAAAGTGTTCTCCTTTTTTAAAAAAAAAGACTGCAGTATTCTAGCTCTTCCATTCTCACATGGTCTGCTACTTCATCTTAGTCAGAGCTTTAGTTGTTAATGGCTCATTGTAAGCTTTATCTTCACTTTCCCTAGTGATATAATATTTGTTCTTGATTGTACAGTTTCTTTGTAATGGATTTGTCTGTTTAAAAACTATTTTATTCTATTCTAGCAAATGGTTATATGTGGAAAGGTGATATGGGGGTTTGAGTGTGTTTCTTAAAAAGCTAATAAGATTGCTAATCATACCCCACACAGATTAAACGTTTAACTAATGGTGATTGACATTTTCTCTATAATGGTGATTTTCCATGTTGGGTAGCTATTGGAGCTTTTTGGTACTGTCAGAGAAACATCCACACACTCTTGCTTTCCTTCTCCTCTTCCTCTGCCTTTCTTAGTTTGCTGCATTTGGGTTTCAAATGCTGAGACTTGAACTGGTTTCGGTCAAGTCTTTTCAGTGTTGGATCTGATTCTTTGTACTAGGCCTCAATTGTGTTTGTCACTTTTCTCATCTTGGTTTCTGGGTTTTAAGGTTTCCTGCTATGTTACTGTTGTTTAGTGGTTTTAGACCTGAAATGGGGTTTTAGAATCTCAATCCTTGTAGATTTCTCTGTCATATTGAGGAATTTCAATCCTTTTGGTTCTGTACTTTATTTTAGTTCAGTTGAGAGGTCAAACATGGCTCCAAGTTTAGATTTTTCTAGGTGGTGGGGAAAGGACAACAATTCAAGTAAGGGTACTCCAGTGGTGGTGACTATGGAGAACCCAAATTACTCTGTACTTGAGATAAATGGTCCTGATGAAGTGTTCAGGCCAGTTGATAAGGACAGAGGCAAGAATGCCAAGCAGTTTACATGGGTTTTGCTTCTCAAGGCCCATAAAGCTGTTGGCTGTGTTGCTTGGCTTGGAAATGTTCTCTGGGACTTGCTTGGAGCCATTAAGAAAAGATTGGTTTTTGGGCAAATGGAGAATGAGAAATCTGGCAAAGGAATCTTGTATAGGGTCATTATGGGGTTCTTAGTGATGGCTTTGGCTTTTCTGGCTTTTGAAGTGGTTGCTCACTTGAAAGGTTGGCATTATTTTGAGAACAGCAGCTTACATATCCCTGAGACTCTGGAGATTAGGGGGTGGCTTCACTTGGTTTATGTGGCATGGTTGGAGTTCAGAGCTGACTGCATTGCACCTGCAATTCAAGCTTTGACGAACTTCTGTATTGCTCTCTTCTTAATCCAATCCGCAGACCGTATGCTGCTTTGTTTAGGTTGCTTCTGGATCAAGTTCAAGAAGATTAAGCCAAGAATTGAAGAAGTTCCATACAAGCCAGACGATTTGGAAGGTTCAGGTCGTAATTACCCCAAGGTTCTTGTTCAAATTCCAATGTGTAATGAGAGAGAGGTATATGAACAATCTATTTCAGCAGTATGCCAACTTGATTGGCCTAAAGAACGCCTTCTGATTCAAGTTCTAGATGATTCTGATGATGAGAGCATACAGTGGTTAATTAAGGCAGAGGTAACTCAGTGGAGCCAAAAAGGTGTCAATATAATCTACCGGCATCGTTTGGTTAGAACTGGTTATAAAGCTGGGAATCTCAAGTCTGCAATGAATTGTGATTATGTGAAGGACTATGAGTTTGTTGCAATCTTTGATGCTGATTTCCAACCAAACCCTGACTTCCTCAAGCTGACAGTTCCCCATTTTAAGGACAATCCTGAGCTTGGGTTGGTTCAGGCGAGGTGGTCTTTTGTTAACAAGGATGAAAACTTGTTGACACGCCTCCAAAATATCAATTTGTGCTTCCACTTTGAAGTGGAACAGCAGGTTAATGGGGTTTTCCTCAATTTCTTTGGCTTCAATGGTACTGCTGGAGTTTGGAGAATTAAAGCACTTGAAGACTCTGGCGGTTGGCTTGAGCGTACAACAGTAGAAGACATGGATATAGCAGTTCGCGCTCATCTTAATGGGTGGAAGTTCATATTCCTTAATGATGTCAAGGTCCTCTGTGAACTTCCCGAGTCTTACGAAGCTTATAAAAAACAGCAACACCGTTGGCATTCTGGTCCGATGCATCTCTTCCGTTTGTGCCTCCCTGCAATAATTACTTCAAAGATGATGTTCTGGAAAAAGGCAAACTTGATACTACTATTCTTTCTCCTTAGGAAGTTAATCCTCCCATTTTACTCTTTCACATTGTTTTGCATAATACTTCCTTTGACCATGTTTGTCCCCGAAGCTGAGCTATCCATGTGGGTTATATGCTACGTGCCCGTTTTTATGTCATTCATGAACATTCTTCCTTCTCCTAGATCTTTTCCCTTCATTGTCCCCTACCTCCTGTTTGAAAACACAATGTCTGTGACCAAATTCAATGCCATGGTATCTGGGTTGTTCCAGTTGGGGAGCTCATATGAATGGATTGTAACCAAGAAGGCGGGACGATCATCAGAATCTGATCTGTTAGCTGTGGAGGAGAGGGAAACGAAGGCCATGAACCACCCACAACTTCATAGAGGAACTTCAGACAGTGGTCTCTCTGAGCTCAACAAGTTAAAAGAGCATCAAGAAGCTGCTGCTCCTAAACCTCCTGTAAAGAAATTGAACAAGATCTACAAGAAAGAGCTGGCACTCGCTTTCTTACTGCTCACTGCTGCAGGTAGAAGCCTTCTATCAGCTCAAGGGGTACATTTTTACTTCCTACTATTTCAAGGCATATCATTTCTACTGGTCGGTCTTGACCTGATTGGTGAGCAGATGAGCTAG
